# Epithelioid Gastrointestinal Stromal Tumor of Duodenum Mimicking Adenocarcinoma: A Case Report

**DOI:** 10.31729/jnma.6104

**Published:** 2021-12-31

**Authors:** Sansar Babu Tiwari, Shovana Karki

**Affiliations:** 1 Department of Pathology, Mahajargunj Medical Campus, Kathmandu, Nepal

**Keywords:** *epithelioid*, *skeinoid fibres*, *tyrosine kinase*

## Abstract

Gastrointestinal stromal tumor is a mesenchymal tumor of gastro-intestinal tract. This epithelioid type gastrointestinal stromal tumor seen in a 22-year-old male with epigastric pain as a presenting symptom had morphological resemblance to carcinoma. However, the immunohistochemistry profile with CD117 and DOG 1 positivity, combined with AE1/AE3 positivity confirmed the tumor as gastrointestinal stromal tumor. Approximately 95% of the patients with gastrointestinal stromal tumor show CD117 immunoreactivity. The treatment approach of CD117 positivity in gastrointestinal stromal tumor has therapeutic benefit with tyrosine kinase inhibitors. Preoperative Imatinib therapy with complete excision can decrease the disease recurrence. Histopathological examination with immunohistochemical studies help to reach the definite diagnosis.

## INTRODUCTION

Gastrointestinal stromal tumor (GIST) are stromal or mesenchymal neoplasms affecting the gastrointestinal tract typically as a subepithelial neoplasms. They are more common in stomach (two-thirds) and proximal small intestine, but they can occur anywhere in the GI tract, rarely in the mesentery, peritoneum and omentum. Almost 95% of the GISTs express c-kit (CD117), those not expressing it are likely to be of epithelioid type. Here we present a case of 22-year-old male presenting with epigastric pain and USG guided biopsy showed epithelioid cells with few glands formation. However, immunochemistry showed CD117 positivity confirming the diagnosis of epithelioid GIST.

## CASE REPORT

A 22-year male presented with chief complains of pain in the right flank for 15 days. On examination the patient had 3 x 3cm mass in the epigastric region. The patient developed back pain and difficulty standing after admission to the hospital.

Contrast-enhanced computed tomography of the abdomen (CECT) showed heterogeneously enhancing soft tissue density mass in right side of retroperitoneum with breech of 3rd part of duodenum and infiltration into infrarenal inferior venecava; tumoral thrombus in infrarenal inferior enhancing lesion in both lobes of liver; abdominal and retroperitoneal lymphadenopathy; lytic lesion in T11 and T12 vertebra with enhancing soft tissue component in bony central spinal canal from T9 to L1 level causing severe narrowing of the bony central spinal canal at the same level.

USG guided duodenal biopsy was performed and histopathological examination was done.

**Figure 1:**
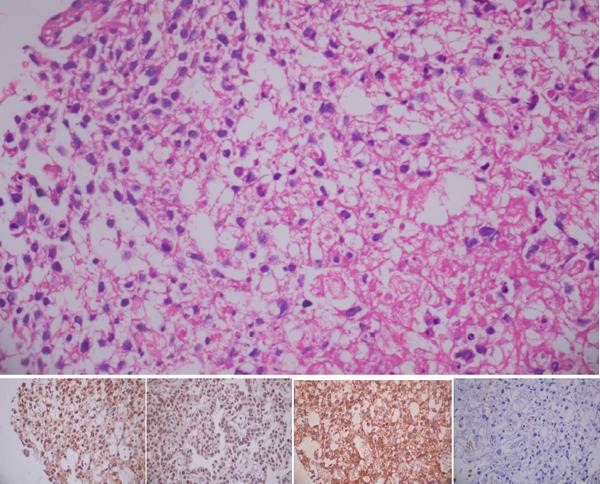
Epithelioid GIST showing CD117, DOG1, PanCK positive and LCA negative.

Microscopically, there was diffuse proliferation of atypical cells with few gland formations. These tumor cells had scant rim of cytoplasm, hyperchromatic nuclei and inconspicuous nucleoli with infrequent mitoses. Stroma showed myxoid change. On immunohistochemistry these tumor cells were C-KIT positive, DOG1 positive, PanCK positive, CK7 negative, CK20 negative and LCA negative (Figure 1).

The patient underwent surgery for resection of the tumor and died after 1 month due to surgical site infection.

## DISCUSSION

GISTs arise from either the interstitial cells of Cajal (ICC) or the precursors of those cells.^1^ The largest density of ICC occurs around the circumference of the myenteric plexus with extension between the inner and outer layers of the muscularis propria.^2^

GISTs are most common in middle-aged and elderly patients. Most of them (approx. 70%) are diagnosed in patients older than 50 years. Almost half of them arise in the stomach (approx. 60%) followed by jejunum and ileum (30%), the duodenum (5%), the colorectum (<5%) and rarely esophagus and appendix.^3^

The most common symptoms are GI bleeding leading to anemia, abdominal pain, nausea, vomiting and weight loss. Some of the GISTs are asymptomatic and are found incidentally at surgery performed for other reasons. In adults, the behavior of GIST can be predicted by anatomic site, tumor size and mitotic activity.^4^ Most of the time metastasis develop within 2 years of diagnosis, the liver and serosa within the abdominal cavity are the usual sites. Rare metastases have been identified in lungs, bone, soft tissue and in few cases intracranial sites.

Most of the GISTs are uninodular whereas multifocal nodules have been found in pediatric population and in GISTs syndrome. It can sometimes ulcerate the overlying mucosa or grow exophytically and protrude toward the serosal aspect. Cut section of these tumors may show areas of hemorrhage, necrosis or cystic change - features that are not indicative of malignancy in GISTs. Histo-morphologically they are grouped into three classes; a) Pure spindle cell, b) Pure epithelioid cell and c) Mixed spindle and epithelioid cells types.^4^ Epithelioid and mixed types are most commonly found in the stomach.

Spindle cell GISTs are composed of uniform, elongated cells with evenly dispersed chromatin, moderate amount of pale to eosinophilic fibrillary cytoplasm and inconspicuous nucleoli. Gastric GISTs frequently show perinuclear vacuoles (due to fixation artefact) that indent the nucleus at one pole. Prominent collagen fibrils (skeinoid fibers) have been described in small bowel GISTs.^5^

Epithelioid GISTs are composed predominantly of cells with either abundant eosinophilic or clear cytoplasm, typically arranged in nests and sheets. The nuclei are round with vesicular chromatin. Scattered multinucleated giant cells, binucleated cells or cells with bizarre nuclei may be present. Stroma may show hyalinization or myxoid change. When epithelioid GISTs involves the mucosa, it may be difficult to differentiate from other epithelioid malignancies.^6^

Almost 95% of GISTs are CD117 (KIT) positive and is considered a sensitive marker, irrespective of its site. It is usually diffuse and pancytoplasmic, however few cases have been described as having membranous and peri-nuclear dotlike staining. Those that are negative for KIT are usually located in the stomach and have epithelioid morphology and contain PDGFRA mutation.^7^ Almost 70% of GISTs express CD34, 20-30% express smooth muscle actin, 5% express S100 and 1-2% are positive for desmin and keratin.

KIT (CD117) has been identified as a receptor tyrosine kinase that plays a role in the development of Interstitial cells of Cajal and binding to its ligand is thought to modulate cell proliferation and inhibit apoptosis. Most of the mutations identified in GIST are activating mutations in KIT (80%) or in PDGFRA (10%).^4^

Discovered on GIST 1 (DOG1) appears to be immunoreactive with GISTs regardless of their KIT/PDGFRA mutational status.^8^ approximately 5% of GISTs lack KIT expression. Inhibition of KIT and PDGFRA by tyrosine kinase inhibitors has revolutionized the treatment of GISTs and demands accurate tumor classification. DOG1.^1^ is a recently described mouse monoclonal antibody reported to have superior sensitivity and specificity compared with KIT (CD117 Small subset of mesenchymal tumors like leiomyosarcoma and synovial sarcoma also show DOG1 positivity.

GISTs with epithelioid features have a list of differential diagnosis that includes melanoma, neuroendocrine tumors and carcinoma. Sometimes, the dedifferentiated GISTs may cause a diagnostic dilemma due to the nuclear pleomorphism that is usually not seen in GISTs. This dedifferentiation can occur de novo or after treatment with selective Tyrosine Kinase Inhibitors and may also show loss of KIT immunoreactivity.

Epithelioid GISTs have some morphological resemblance to epithelial malignancies and pathologists should be aware of this. Immunohistochemistry is of pertinent value in reaching the correct diagnosis.
